# Acidosis significantly alters immune checkpoint expression profiles of T cells from oesophageal adenocarcinoma patients

**DOI:** 10.1007/s00262-022-03228-y

**Published:** 2022-06-16

**Authors:** Maria Davern, Noel E. Donlon, Fiona O’Connell, Caoimhe Gaughan, Cillian O’Donovan, Mohammed Habash, Andrew D. Sheppard, Michael MacLean, Margaret R. Dunne, Jenny Moore, Hugo Temperley, Melissa J. Conroy, Christine Butler, Anshul Bhardwaj, Narayanasamy Ravi, Claire L. Donohoe, John V. Reynolds, Joanne Lysaght

**Affiliations:** 1 Cancer Immunology and Immunotherapy Group, Department of Surgery, Trinity St. James’s Cancer Institute, Trinity Translational Medicine Institute, St. James’s Hospital Campus, Trinity College, Dublin 8, Ireland; 2 Department of Surgery, Trinity St. James’s Cancer Institute, Trinity Translational Medicine Institute, St. James’s Hospital, Trinity College Dublin, Dublin, Ireland

**Keywords:** A2aR, Acidosis, Lactate, Immune checkpoints, Oesophageal adenocarcinoma, Tie-2

## Abstract

Tumour acidosis contributes to cancer progression by inhibiting anti-tumour immunity. However, the effect of acidosis on anti-tumour T cell phenotypes in oesophageal adenocarcinoma (OAC) is unknown. Therefore, this study investigated the effect of acidosis on anti-tumour T cell profiles and if immune checkpoint blockade (ICB) could enhance anti-tumour T cell immunity under acidosis. Acidic conditions substantially altered immune checkpoint expression profiles of OAC patient-derived T cells, upregulating TIM-3, LAG-3 and CTLA-4. Severe acidosis (pH 5.5) significantly decreased the percentage of central memory CD4^+^ T cells, an effect that was attenuated by ICB treatment. ICB increased T cell production of IFN-γ under moderate acidosis (pH 6.6) but not severe acidosis (pH 5.5) and decreased IL-10 production by T cells under severe acidic conditions only. A link between lactate and metastasis was also depicted; patients with nodal metastasis had higher serum lactate levels (*p* = 0.07) which also positively correlated with circulating levels of pro-angiogenic factor Tie-2. Our findings establish that acidosis-induced upregulation of immune checkpoints on T cells may potentially contribute to immune evasion and disease progression in OAC. However, acidic conditions curtailed ICB efficacy, supporting a rationale for utilizing systemic oral buffers to neutralize tumour acidity to improve ICB efficacy.

**Graphical abstract:**

Study schematic—PBMCs were isolated from OAC patients (A) and expanded ex vivo for 7 days using anti-CD3/28 +IL-2 T cell activation protocol (B) and further cultured for 48 h under increasing acidic conditions in the absence or presence of immune checkpoint blockade (nivolumab, ipilimumab or dual nivolumab + ipilimumab) (C). Immunophenotyping was then carried out to assess immune checkpoint expression profiles and anti-tumour T cell phenotypes (D). Serum lactate was assessed in OAC patients (E–F) and levels were correlated with patient demographics (G) and the levels of circulating immune/pro-angiogenic cytokines that were determined by multiplex ELISA (H). Key Findings—severe acidic conditions upregulated multiple immune checkpoints on T cells (I). Efficacy of ICB was curtailed under severe acidic conditions (J). Circulating lactate levels positively correlated with circulating levels of pro-angiogenic factor tie-2 and higher serum lactate levels were found in patients who had nodal metastasis (K). 
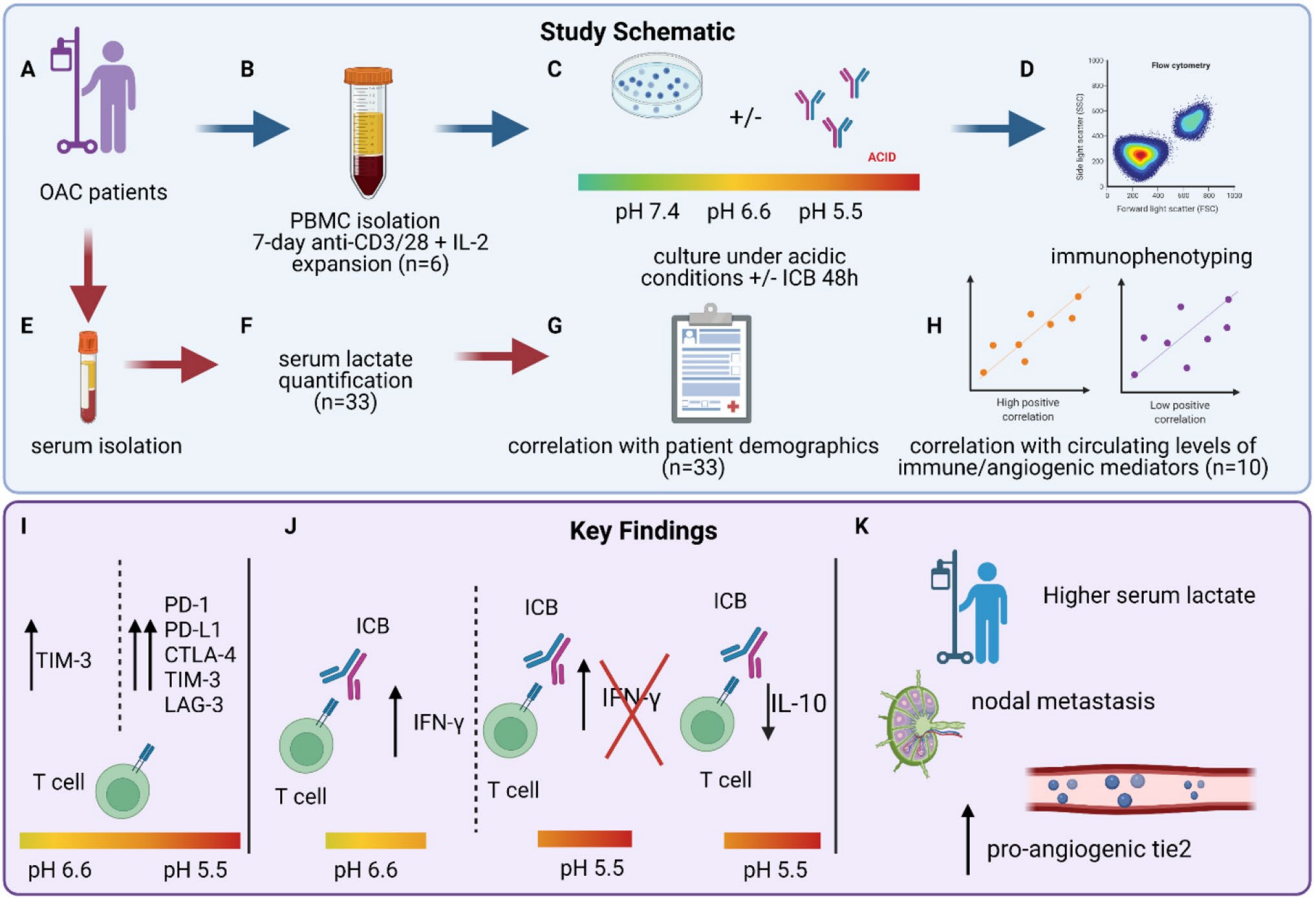

**Supplementary Information:**

The online version contains supplementary material available at 10.1007/s00262-022-03228-y.

## Introduction

Tissue acidosis (pH 6.0–7.0) is typically accompanied with inflammatory processes in peripheral tissues, where the pH can decrease as low as 5.5 [1]. Moreover, acidosis is a hallmark feature of solid tumours, which range in pH from 5.7 to 7.0 [2, 3]. A combination of poor tissue perfusion and high rates of lactic acid due to fermentative glycolytic metabolism contributes to the generation of an acidic tumour microenvironment [4]. This acidity also contributes to tumour progression by substantially altering both the innate and adaptive immune responses [5]. Overall, a low pH has been shown to suppress anti-tumour immunity and promote the pro-tumourigenic actions of the immune system [6]. However, extracellular acidosis can either stimulate or suppress innate immune responses depending on both the cell type involved and the particular response analysed [6].

Acidosis delays neutrophil apoptosis [7], promoting their differentiation into a pro-angiogenic profile [8]. However, it has been suggested that a high concentration of protons as a consequence of a low pH might be recognized by innate immune cells as a danger-associated molecular pattern [1]. Extracellular acidosis (pH 6.5) has been shown to increase the endocytic activity of dendritic cells (DCs) and their expression of MHC class II, CD40, and CD86 [9]. Moreover, DCs pulsed with antigens at low pH values showed an improved efficacy to induce specific cytotoxic responses mediated by CD8^+^ T cells as well as specific antibody responses in vivo [9]. In human DCs derived from monocytes cultured with IL-4 and GM-CSF, transient exposure to pH 6.5 conditions markedly increases the expression of HLA-DR, CD40, CD80, CD86, CD83, and CCR7, improves the T cell priming ability of DCs, and increases the production of IL-12, stimulating the synthesis of IFN-*γ*, but not IL-4, by antigen-specific CD4^+^ T cells [10]. Of particular note, low concentrations of LPS abrogated DC maturation induced by pH 6.5 [10], suggesting a cross-talk between the activation pathways triggered by extracellular protons and LPS in DCs. Similarly, Tong et al*.* further confirmed the ability of extracellular acidosis to induce the phenotypic maturation of human DCs via activation of acid-sensing ion channels (ASIC) receptors, a family of proton receptors [11].

In contrast, a large body of evidence indicates that low pH has been shown to suppress T cell-mediated immunity [1]. Low pH has been shown to suppress the cytotoxic response mediated by CD8^+^ T cells as well as the production of IFN-*γ* by Th1 cells [12]. Calcinotto et al*.* have shown that lowering the environmental pH to values of 6.0–6.5 induced an anergic state in human and mouse tumour-specific CD8^+^ T cells, characterized by impaired cytotoxic activity, inhibition of cytokine production, reduced expression of the alpha chain of the IL-2R (CD25), and a diminished activation of extracellular signal-regulated kinase (ERK) and STAT5 upon T cell activation [13]. However, buffering the pH to neutral values restored T cell function [13, 14]. Moreover, systemic treatment of tumour-bearing mice with proton pump inhibitors improved the therapeutic efficacy of immunotherapy, suggesting that proton pump inhibitors might represent useful therapeutic tools to reverse the anergy of tumour-infiltrating T cells and to improve the performance of immunotherapy approaches used in cancer [15].

However, the effect of acidosis on anti-tumour T cell phenotypes in the context of oesophageal adenocarcinoma (OAC) remains unknown. Therefore, this study examines the direct effects of acidosis (pH 5.5, 6.6 and 7.4) on OAC patient blood-derived T lymphocyte function and the ability of immune checkpoint blockade (ICB) to enhance T cell immunity under acidic conditions reflective of the tumour microenvironment. We also assess systemic lactate levels in OAC patients post-chemo (radio)therapy treatment prior to surgery and correlate these levels with clinical characteristics to provide a deeper insight into how serum lactate levels may be used as a clinical indicator of treatment response and prognosis. Importantly, systemic lactate levels are also correlated with circulating levels of pro-tumourigenic and anti-tumourigenic immune mediators as well as pro-angiogenic factors to shed light on potential pro-tumourigenic processes that may be interlinked with the lactate axis.

## Methods

### Ethical approval

This study was carried out in accordance with the World Medical Association’s Declaration of Helsinki guidelines on medical research involving human subjects. Patients provided informed consent for sample and data acquisition, and the study received full ethical approval from the St James’s Hospital/AMNCH Ethical Review Board. Patient samples were pseudonymised to protect the privacy rights of the patients in line with GDPR and data protection policies.

### Specimen collection

All patients involved in this study were enrolled from 2018 to 2021. Treatment-naïve whole blood was obtained from OAC patients undergoing endoscopy at St James’s Hospital at time of diagnosis, prior to initiation of chemotherapy or radiotherapy. The group consisted of 21 males and 12 females, with an average age of 65.8 years. The patient demographics are detailed in Tables 1 and 2.

**Table 1:** Patient demographic table

Age (mean years)	65.8
Sex ratio (M:F)	21:12
Clinical tumour stage (no. patients)	
T0	0
T1	9
T2	8
T3	16
T4	0
Clinical nodal status (no. patients)	
Positive	16
Negative	17

**Table 2:** Patient demographic table for cohort of six OAC patients recruited for profiling the effects of acidity on T cell phenotype, function and efficacy of ICB blockade ex vivo

Age (mean years)	64.5
Sex ratio (M:F)	4:2
Clinical tumour stage (no. patients)	
T0	0
T1	1
T2	2
T3	3
T4	0
Clinical nodal status (no. patients)	
Positive	4
Negative	2

### Cell culture and treatment

Treatment-naïve OAC donor peripheral blood mononuclear cells (PBMCs) (*n* = 6) were isolated from whole blood using density gradient centrifugation and expanded for 7 days with plate bound anti-CD3 (10 μg/ml, Biolegend, USA), anti-CD28 (10 μg/ml, Ancell, USA) and recombinant human IL-2 (100 units/ml, Immunotools, Germany) for 7 days in complete RPMI 1640 medium (cRPMI) (containing 2 mM L-glutamine (Gibco) and supplemented with 1% (v/v) penicillin–streptomycin (50 U/ml penicillin 100 μg/ml streptomycin) and 10% (v/v) foetal bovine serum (Gibco)). PBMCs were seeded in cRPMI at increasing levels of acidity (pH 7.4, 6.6 and 5.5) in the absence or presence of nivolumab (10 μg/ml), ipilimumab (10 μg/ml) or dual nivolumab and ipilimumab (10 μg/ml and 10 μg/ml, respectively) at 37 °C 5% CO_2_ for an additionally 48 h. Acidity was altered using a pH meter and concentrated hydrochloric acid (1 molar, Sigma Aldrich, USA). Wu et al. [16] demonstrated that the acidic pH present in lymph nodes does not hinder initial activation of naïve T cells. Typically, T cells spend 5–7 days in the lymph node undergoing activation before travelling to the tumour site; therefore, in this study, a 7-day T cell activation timepoint was chosen followed by an additional 48 h culture in acidic conditions to recapitulate the timing of human biological processes.

### OE33 cell line culture and treatment

Human OE33 cells were purchased from European Collection of Cell Cultures. OE33 cells were grown in cRPMI and maintained in a humidified chamber at 37 °C 5% CO_2_. OE33 cells were seeded at a density of 0.1 × 10^6^ cells/ml in cRPMI in a 12 well plate and left adhere overnight. The following day the media was replaced with cRPMI at increasing levels of acidity (pH 7.4, 6.6 and 5.5). The acidity was altered using a pH meter and concentrated hydrochloric acid. Cell lines were tested regularly to ensure mycoplasma negativity. The OE33 cell line was established from a poorly differentiated stage IIA adenocarcinoma of the lower oesophagus (Barrett’s metaplasia) of a 73-year-old female patient.

### Flow cytometry staining

Trypsinised OE33 cells or OAC donor PBMCs were stained with Zombie Aqua™, Violet™ or NIR™ viability dye (Biolegend, USA). Antibodies used for staining included LAG-3-FITC, CD160-PerCPCy5.5, PD-1-PE/Cy7, TIGIT-PE/Cy7, CD45RA-PE/Cy7, CD45RO-BV510, CD3-APC, CD3-PerCP, CD4-BV510, CD4-APC (Biolegend, USA), CD69-PE, CD62L-FITC, CD8-BV421 (BD Biosciences, USA), CD27-APEeFluor780 (eBioscience, USA), TIM-3-AF647, CTLA-4-PE/Cy5, KLRG-1-APC, PD-L1-FITC, PD-L2-PE (BD Bioscience, USA), A2aR-PE (Bio-techne, USA). PBMCs were resuspended in FACs (PBMS, 0.05% sodium azide, 1% foetal bovine serum) buffer and acquired using BD FACs CANTO II (BD Biosciences) using Diva software and analysed using FlowJo v10 software (TreeStar Inc.). Gating strategies for IC analysis and activation marker analysis are shown in Figs. S1 and S2, respectively.

For intracellular cytokine staining, PBMCs were treated with PMA (10 ng/ml) and ionomycin (1 µg/ml) for the last 4 h of the incubation. Anti-CD107a-PE (BD Biosciences, USA) was added during stimulation. For the last 3 h of the incubation, PBMCs were treated with brefeldin A (10 µg/ml, eBiosciences). Cells were harvested and washed in FACs buffer, and intracellular cytokines were assessed using a Fixation/Permeabilisation kit (BD Biosciences), as per manufacturer’s recommendations. Briefly, cells were stained with cell surface antibodies (CD8-BV421, CD3-APC or CD3-PerCP, CD4-PerCP, CD4-APC or CD4-BV510 (Biolegend, USA)) washed, permeabilised, and then stained for intracellular cytokines: IFN-γ-BV510, IL-4-PE/Cy7, IL-10-PE (Biolegend, USA) and TNF-α-APC (BD Biosciences, USA). Cells were resuspended in FACs buffer and acquired using BD FACs CANTO II (BD Biosciences). Supplemental Table 1 contains all the flow cytometry panels used in these experiments.

### Lactate assay

Lactate concentration was assessed in OAC donor serum samples (*n* = 33) using the lactate colorimetric/fluorometric assay kit purchased from BioVision (Catalog#K607-100). The assay was carried out as per Manufacturer’s instructions. In brief, 10 µl of each serum sample was added to a flat bottomed 96-well plate in duplicate and 40 µl of lactate assay buffer was added to each sample. The lactate standards were prepared using assay buffers from the kit to generate the standard curve. The standard curve wells were adjusted to a total volume to 50 µl/well with lactate assay buffer. 50 µl reaction mix was added to each well and mixed well. A background control was also prepared which contained 98 μl of lactate assay buffer and 2 μl of probe. Each reaction well was prepared in duplicate. The plate was incubated for 30 min at room temperature and protected from light, and the absorbance was measured at 570 nm using the Versa Max microplate reader (Molecular Devices, Sunnyvale, CA, USA).

### Collection of serum

Whole blood was collected using vacutainer tubes suitable for collecting serum (BD Biosciences). Tubes were centrifuged at 3000 RPM for 10 min at room temperature, and serum was collected and stored at − 80 °C to be used later for experimentation.

### Quantification of serum immune proteins

Serum was prepared according to the manufacturer’s instructions (Meso Scale Diagnostics, USA). To assess angiogenic, vascular injury, pro-inflammatory, cytokine, chemokine and soluble checkpoint secretions from the serum, a 54-plex ELISA kit was used (Meso Scale Diagnostics, USA). The multiplex kit was used to quantify the secretions of CRP, Eotaxin, Eotaxin-3, FGF(basic), Flt-1, GM-CSF, ICAM-1, IFN-γ, IL-10, IL-12/IL-23p40, IL-12p70, IL-13, IL-15, IL-16, IL-17A, IL-17A/F, IL-17B, IL-17C, IL-17D, IL-1RA, IL-1α, IL-1β, IL-2, IL-21, IL-22, IL-23, IL-27, IL-3, IL-31, IL-4, IL-5, IL-6, IL-7, IL-8, IL-8 (High Abundance), IL-9, IP-10, MCP-1, MCP-4, MDC, MIP-1α, MIP-1β, MIP-3α, PlGF, SAA, TARC, Tie-2, TNF-α, TNF-β, TSLP, VCAM-1, VEGF-A, VEGF-C and VEGF-D and immune checkpoints PD-1, PD-L1, TIGIT, TIM-3, CD276 and CD80. All assays were run as per manufacturer’s instructions, with an overnight supernatant incubation protocol used for all assays except Angiogenesis Panel 1 and Vascular Injury Panel 2, which were run according to the same day protocol.

### Statistical analysis

Data were analysed using GraphPad Prism (GraphPad Prism, San Diego, CA, USA) software and were expressed as mean ± SEM. Statistical differences between treatments within cancer donors or healthy donors were analysed using paired non-parametric *t*-test, and statistical differences between treatments between healthy donors and cancer donors were analysed using unpaired non-parametric *t*-tests. Statistical significance was determined as *p* ≤ 0.05. Spearman correlations were performed to analyse correlation data between clinical characteristics and flow data. tSNE plots were generated using FlowJo software version 10.8.1.

## Results

### Acidic conditions substantially alter IC expression profiles of T cells but have minimal effect on IC expression profiles of OAC cells

**Fig. 1:**
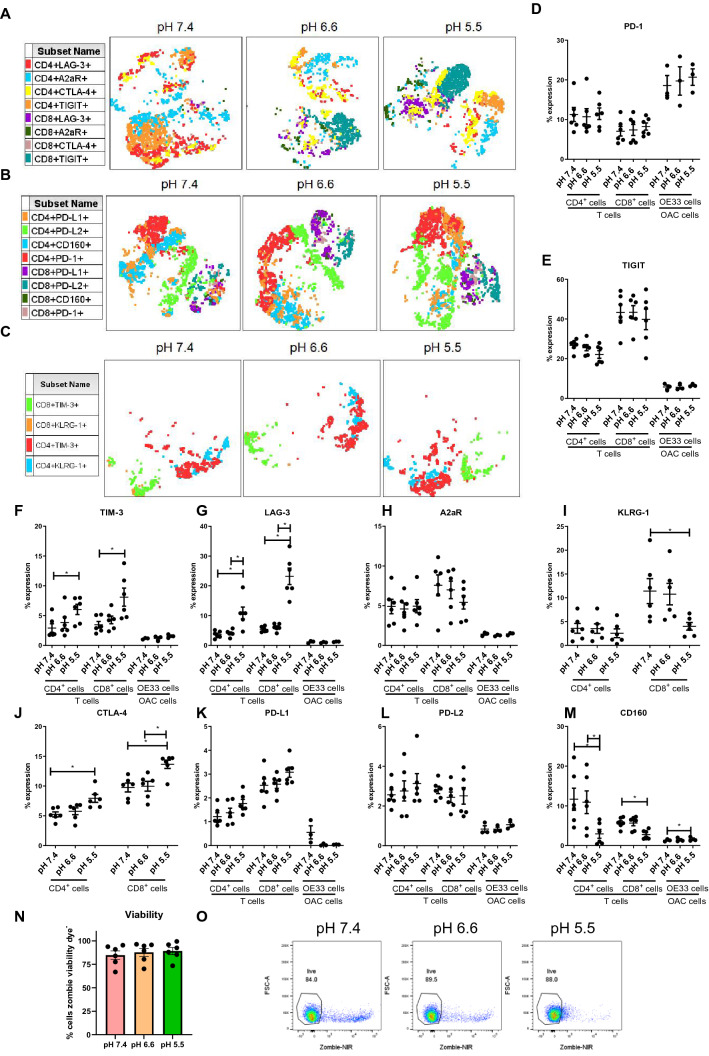
Acidic conditions upregulated TIM-3, LAG-3 and CTLA-4 and decreased KLRG-1 and CD160 ICs on OAC patient-derived T cells. PBMCs were isolated from peripheral blood of treatment-naïve OAC patients (*n* = 6) and expanded ex vivo for 7 days in the presence of plate bound anti-CD3/anti-CD28 and recombinant human IL-2. Following a 7-day expansion, PBMCs were cultured for 48 h in media with increasing levels of acidity (pH 7.4, pH 6.6 and pH 5.5). Subsequently, IC expression profiles were assessed on the surface of viable CD3^+^, CD3^+^CD4^+^ and CD3^+^CD8^+^ cells by flow cytometry. OE33 cells were cultured for 48 h in media with increasing levels of acidity (pH 7.4, pH 6.6 and pH 5.5), and IC expression profiles were assessed by flow cytometry. **A**–**C** tSNE plots showing the effect of acidosis on the frequency of CD4^+^ and CD8^+^ cells expressing ICs on viable CD3^+^ singlet cells. **D**–**M** effect of acidosis on the frequency of CD4^+^ and CD8^+^ cells expressing ICs. **N** viability of cells within the lymphogate as zombie negative with representative dot plots shown in (**O**). Expression presented as percentages ± SEM. Paired, non-parametric *t*test for PBMCs (*n* = 6) and paired parametric t test for OE33 cells (*n* = 3), *p < 0.05

Firstly, this study profiled the effect of acidosis on IC expression profiles of T cells and OAC cells. A well-documented immune evasion strategy employed by tumour cells is their upregulation of ICs on the surface of tumour cells which promotes T cell dysfunction via binding to cognate receptors on T cell surfaces inducing immune exhaustion. Interestingly, the findings from this study demonstrated that acidosis has a minimal effect on IC expression profiles of OAC cells. Basally, ICs were expressed at very low levels on OAC cells or were absent with the exception of PD-1 which was more widely expressed (Fig. 1). However, acidosis substantially alters IC expression on T cell surfaces (Fig. 1).

tSNE plots for PBMCs cultured in media with increasing levels of acidity are showcased in Fig. 1A–C which provide a visual presentation of the spatial distribution of IC expression profiles for CD4^+^ and CD8^+^ T cells and how they cluster together in a one two-dimensional plots. TIM-3, LAG-3 and CTLA-4 expression was significantly increased on the surface of CD4^+^ T cells and CD8^+^ T cells when cultured in pH 5.5 cRPMI compared with pH 7.4 cRPMI (Fig. 1F, G, J). Similarly, the expression of LAG-3 was significantly increased on the surface of CD4^+^ T cells and CD8^+^ T cells in pH 5.5 cRPMI compared with pH 6.6 cRPMI (Fig. 1G). CTLA-4 was significantly increased on the surface of CD8^+^ T cells in pH 5.5 cRPMI compared with pH 6.6 cRPMI (Fig. 1J).

KLRG-1 was significantly decreased on the surface of CD8^+^ T cells when cultured in pH 5.5 cRPMI compared with pH 7.4 cRPMI (Fig. 1I). The expression of CD160 significantly decreased on the surface of CD4^+^ T cells when cultured in pH 5.5 cRPMI compared with pH 6.6 cRPMI and pH 7.4 cRPMI (Fig. 1M). Similarly, the expression of CD160 significantly decreased on the surface of CD8^+^ T cells when cultured in pH 5.5 cRPMI compared with pH 7.4 cRPMI (Fig. 1M). In contrast, the expression of CD160 was significantly increased on the surface of OE33 cells when cultured in pH 5.5 cRPMI compared with pH 7.4 cRPMI (*p* = 0.03) (Fig. 1M). PD-1 (Fig. 1D), TIGIT (Fig. 1E), A2aR (Fig. 1H), PD-L1 (Fig. 1K) and PD-L2 (Fig. 1L) expression on T cells was not affected by acidosis. Figure 1N–O illustrates the effect of acidosis on the viability of CD4^+^ and CD8^+^ T cells.

Overall, acidosis significantly altered expression profiles of ICs on the surface of OAC-derived T cells. LAG-3, TIM-3 and CTLA-4 were significantly increased, and KLRG-1 and CD160 were significantly decreased on the surface of T cells under acidic conditions.

**Fig. 2:**
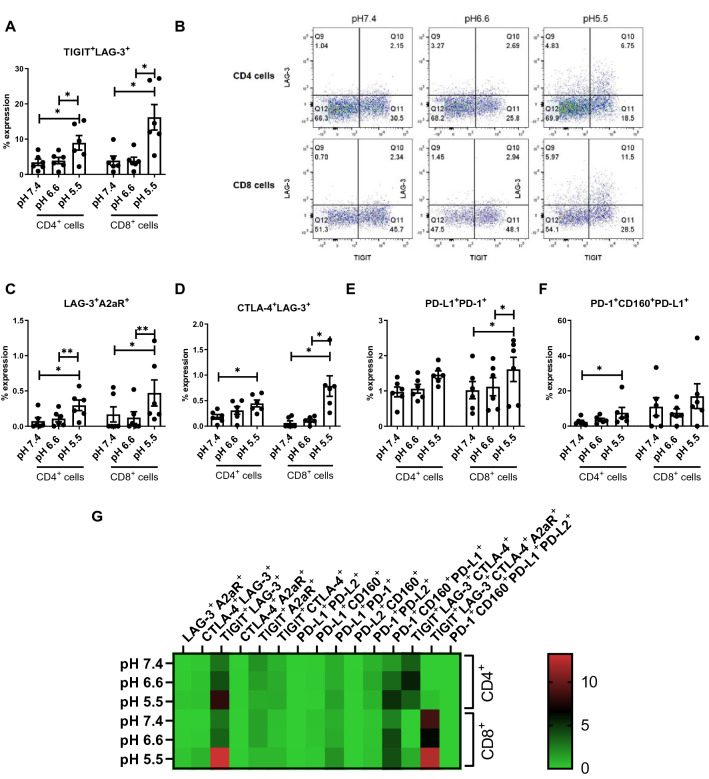
Effect of acidic conditions on the co-expression of ICs on the surface of OAC patient-derived T cells. PBMCs were isolated from peripheral blood of treatment-naïve OAC patients (*n* = 6) and expanded ex vivo for 7 days in the presence of plate bound anti-CD3/anti-CD28 and recombinant human IL-2. Following a 7-day expansion, PBMCs were cultured for 48 h in media with increasing levels of acidity (pH 7.4, pH 6.6 and pH 5.5). CD3^+^CD4^+^ and CD3^+^CD8^+^ cells were stained with zombie viability dye and antibodies specific for IC ligands and receptors, and co-expression of multiple ICs was assessed by flow cytometry (**A**–**K**). **B** displays representative dot plots showing the effect of acidosis on the expression of LAG-3^+^TIGIT^+^CD4^+^ and LAG-3^+^TIGIT^+^CD8^+^ cells. **L** displays a heatmap summarising the effect of acidosis on the co-expression of multiple IC ligands and receptors on CD4^+^ and CD8^+^ T cells. Expression presented as percentages ± SEM. Paired, non-parametric t test for PBMCs (*n* = 6) and paired parametric t test for OE33 cells (*n* = 3)

The effect of acidosis was also assessed on the co-expression of multiple ICs on T cells, and similar results were observed in which ICs were co-expressed and significantly upregulated on CD4^+^ and CD8^+^ T cells (Fig. 2). The frequency of TIGIT^+^LAG-3^+^ (Fig. 2A, B), LAG-3^+^A2aR^+^ (Fig. 2C), CTLA-4^+^LAG-3^+^ (Fig. 2D) expressing CD4^+^ and CD8^+^ T cells was significantly increased under pH 5.5 conditions compared with pH 7.4 conditions. Similarly, the frequency of PD-L1^+^PD-1^+^ (Fig. 2E) CD8^+^ T cells was significantly increased under pH 5.5 conditions compared with pH 7.4 conditions. The frequency of PD-1^+^CD160^+^PD-L1^+^ (Fig. 2F) CD4^+^ T cells was significantly increased under pH 5.5 conditions compared with pH 7.4 conditions. Figure 2G summarises the effects of increasing acidic conditions on the co-expression of multiple ICs on T cells. Figure S3A–E details graphs depicting the effect of acidosis on the co-expression of ICs on T cells that were not significantly altered.

### Acidic conditions decrease the frequency of central memory T cells which is abrogated by ICB

**Fig. 3:**
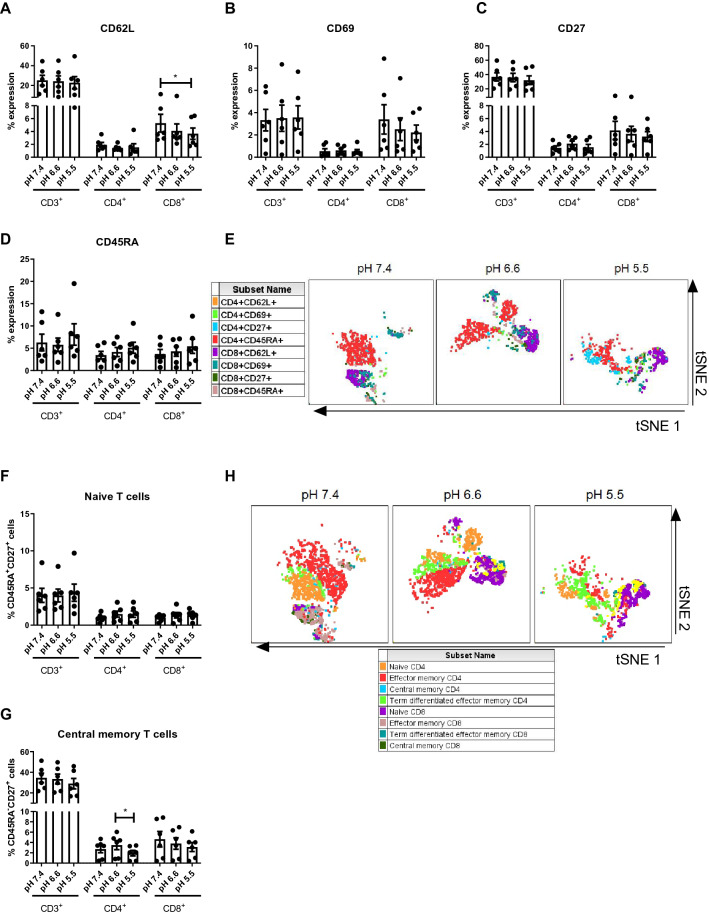
The percentage of central memory CD4^+^ T cells decreases under acidic conditions. PBMCs were isolated from peripheral blood of treatment-naïve OAC patients (*n* = 6) and expanded ex vivo for 7 days in the presence of plate bound anti-CD3/anti-CD28 and recombinant human IL-2. Following a 7-day expansion, PBMCs were cultured for 48 h in media with increasing levels of acidity (pH 7.4, pH 6.6 and pH 5.5). Expression of markers reflective of T cell activation status was assessed on viable CD3^+^ cells, CD3^+^CD4^+^ and CD3^+^CD8^+^ cells by flow cytometry. Activation markers assessed included: CD62L (**A**), CD69 (**B**), CD27 (**C**) and CD45RA (**D**). tSNE plots depict the spatial distribution of CD4^+^ and CD8^+^ T cells expressing different activation markers under increasing conditions of acidosis on viable CD3^+^ singlet cell population (**E**). T cell differentiation status was also assessed, and the percentage of viable naïve (CD45RA^+^CD27^+^) (**F**), central memory (CD45RA^−^CD27^+^) (**G**), effector memory (CD45RA^−^CD27^−^) (**H**) and terminally differentiated effector memory (CD45RA^+^CD27^−^) (**I**) CD3^+^, CD3^+^CD4^+^ and CD3^+^CD8^+^ cells were determined by flow cytometry. tSNE plots presenting the spatial distribution of CD4^+^ and CD8^+^ T cell differentiation states under increasing conditions of acidosis on the viable CD3^+^ singlet cell population (**J**). Paired, non-parametric t test, *p < 0.05. Expression presented as percentages ± SEM

Given that acidosis significantly altered the expression profile of ICs on T cell surfaces we sought to investigate if acidosis might alter the activation status or differentiation status of T cells (Fig. 3).

**Fig. 4:**
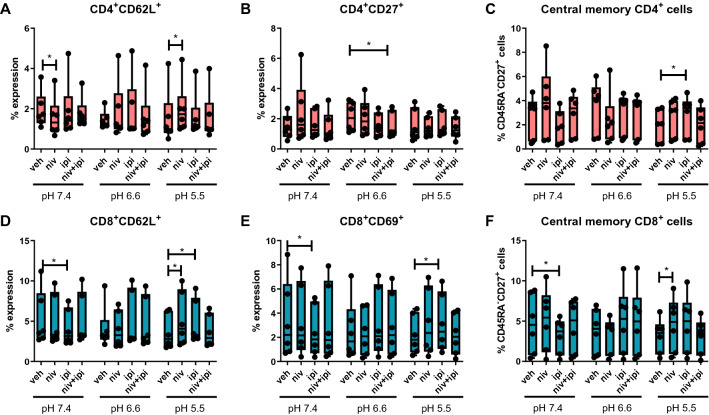
ICB increases the percentage of central memory T cells under acidic conditions. PBMCs were isolated from peripheral blood of treatment-naïve OAC patients (*n* = 6) and expanded ex vivo for 7 days in the presence of plate bound anti-CD3/anti-CD28 and recombinant human IL-2. Following a 7-day expansion, PBMCs were cultured for 48 h in media with increasing levels of acidity (pH 7.4, pH 6.6 and pH 5.5) in the absence or presence of ICB. ICB included nivolumab (niv), ipilimumab (ipi) or dual nivolumab-ipilimumab (niv + ipi). Expression of markers reflective of T cell activation status was assessed on viable CD3^+^CD4^+^ (**A**–**C**) and CD3^+^CD8^+^ (**D**–**F**) cells by flow cytometry (*n* = 6). Markers assessed included: CD62L, CD69, CD27 and CD45RA. The percentage of viable naïve (CD45RA^+^CD27^+^), central memory (CD45RA^−^CD27^+^), effector memory (CD45RA^−^CD27^−^) and terminally differentiated effector memory (CD45RA^+^CD27^−^) CD3^+^CD4^+^and CD3^+^CD8^+^cells was also determined by flow cytometry. Only significant changes are shown in the figure, and non-significant changes are outlined in Fig. S4. Dead cells were excluded using a zombie viability dye. Paired, non-parametric t test, *p < 0.05. Expression presented as percentages ± SEM

Acidity did not alter the expression of T cell activation markers on CD4^+^ T cells, and there were no significant changes in the expression of CD62L (Fig. 3A), CD69 (Fig. 3B), CD27 (Fig. 3C) or CD45RA (Fig. 3D) on the surface of CD4^+^ T cells under acidic conditions compared with neutral acidity. However, acidity did alter the expression of one T cell activation marker on CD8^+^ T cells, and the frequency of CD8^+^ T cells expressing CD62L was significantly decreased when cultured in pH 5.5 cRPMI compared with cells cultured in pH 7.4 cRPMI (*p* = 0.03) (Fig. 3A). Furthermore, acidic conditions did not alter the expression of CD69 (Fig. 3B), CD27 (Fig. 3C) or CD45RA (Fig. 3D) on the surface of CD8^+^ T cells compared with neutral acidity. Figure 4E details tSNE plots which shows the spatial distribution of CD4^+^ and CD8^+^ T cells expressing different activation markers under increasing conditions of acidosis.

The frequency of central memory CD4^+^ T cells was significantly decreased when cultured in pH 5.5 cRPMI compared with cells cultured in pH 6.6 cRPMI (*p* = 0.03) (Fig. 3G). In contrast, acidosis did not affect the frequency of central memory CD8^+^ T cells (Fig. 3G). Furthermore, acidic conditions did not significantly alter the frequency of naïve (Fig. 4F), effector memory (Fig. S4A), or terminally differentiated (Fig. S4B) CD4^+^ T cells and CD8^+^ T cells. Figure 4H showcases tSNE plots depicting the spatial distribution of CD4^+^ and CD8^+^ T cell differentiation states under increasing conditions of acidosis.

Overall, acidosis did not have substantial effects on T cell activation status or differentiation state. However, acidosis did marginally but significantly decrease CD62L expression on the surface of CD8^+^ T cells and significantly decrease the frequency of CD4^+^ central memory T cells ex vivo*.*

To further understand how ICB might improve T cell function under acidic conditions, which are typically found within the tumour microenvironment, single agent nivolumab, ipilimumab or dual nivolumab and ipilimumab treatment were added to the acidic ex vivo culture system (Fig. 4). In pH 7.4 cRPMI nivolumab significantly decreased CD62L expression on the surface of CD4^+^ T cells compared with untreated cells in pH 7.4 cRPMI (Fig. 4A). In contrast, under culture conditions of pH 5.5 cRPMI CD62L expression is significantly increased on the surface of CD4^+^ T cells following nivolumab treatment compared with untreated cells (Fig. 4A). Moreover, dual nivolumab and ipilimumab treatment significantly decreased CD27 expression on the surface of CD4^+^ T cells compared with untreated cells in pH 6.6 cRPMI only (Fig. 4B). Ipilimumab significantly increased the frequency of central memory CD4^+^ T cells compared with untreated cells in pH 5.5 cRPMI acidic conditions only (Fig. 4C). Ipilimumab significantly decreased CD62L expression on the surface of CD8^+^ T cells compared with untreated cells in pH 7.4 cRPMI (Fig. 4D). In contrast, single-agent nivolumab and single-agent ipilimumab significantly increased CD62L expression on the surface of CD8^+^ T cells compared with untreated cells in pH 5.5 cRPMI (Fig. 4D). In addition, under culture conditions of pH 7.4, ipilimumab significantly decreased CD69 expression on the surface of CD8^+^ T cells compared with untreated cells (Fig. 4E). However, under pH 5.5 cRPMI culture conditions, ipilimumab significantly increased CD69 expression on the surface of CD8^+^ T cells compared with untreated cells in pH 5.5 cRPMI (Fig. 4E). In pH 7.4 cRPMI, single-agent ipilimumab significantly decreased the frequency of central memory CD8^+^ T cells compared with untreated cells in pH 7.4 cRPMI (Fig. 4F). However, in pH 5.5 cRPMI single-agent nivolumab significantly increased the frequency of central memory CD8^+^ T cells compared with untreated cells (Fig. 4F). Figure S4 showcases the graphs depicting the non-significant effects of ICB on T cell activation marker expression and T cell differentiation status.

Overall, single-agent ipilimumab treatment marginally but significantly increased the frequency of central memory-like CD4^+^ T cells under severe acidic conditions (pH 5.5) ex vivo. Single-agent ipilimumab marginally but significantly decreased the frequency of central memory-like CD8^+^ T cells under neutral pH 7.4 conditions. In contrast, single-agent nivolumab marginally but significantly increased and single-agent ipilimumab increased the frequency of central memory-like CD8^+^ T cells under severe acidic conditions (pH 5.5).

### ICB enhances anti-tumour cytokine profiles of T cells under acidic conditions

**Fig. 5:**
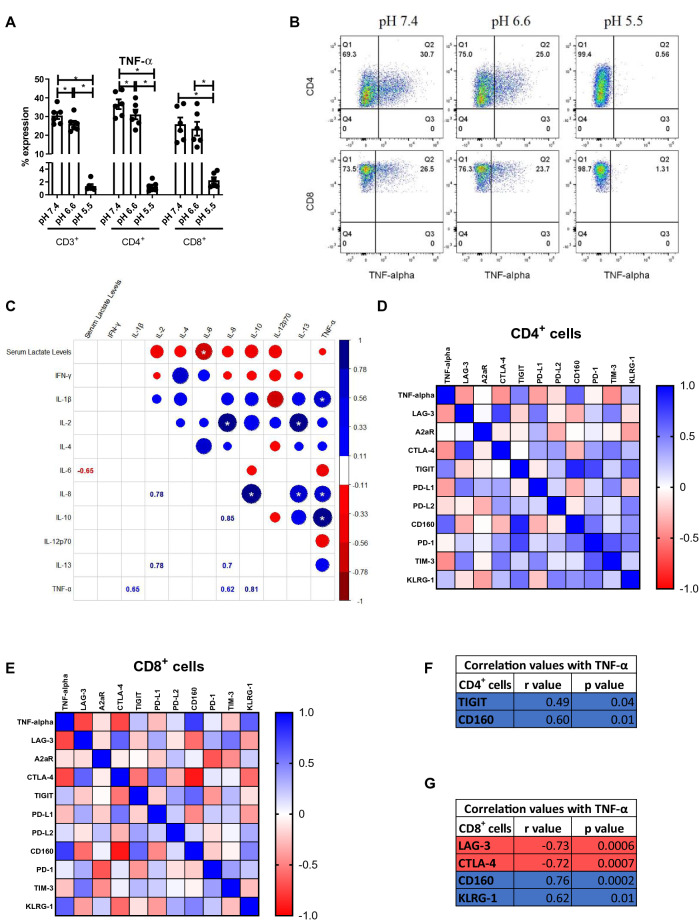
The percentage of TNF-α producing T cells is significantly decreased under acidic conditions ex vivo. PBMCs were isolated from peripheral blood of treatment-naïve OAC patients (*n* = 6) and expanded ex vivo for 7 days in the presence of plate bound anti-CD3/anti-CD28 and recombinant human IL-2. Following a 7-day expansion, PBMCs were cultured for 48 h in media with increasing levels of acidity (pH 7.4, pH 6.6 and pH 5.5). Intracellular staining was conducted to assess CD3^+^, CD3^+^CD4^+^ and CD3^+^CD8^+^ cell production of TNF-α (**A**–**B**), IFN-γ (Fig. S5A), IL-10 (Fig. S5B.) and IL-4 (Fig. S5C) cytokines by flow cytometry. The cytotoxic potential of CD3^+^CD8^+^ T cells was also assessed using a CD107a degranulation assay by flow cytometry (Fig. S5D). Paired, non-parametric t test, *p < 0.05. Expression presented as percentages ± SEM. **C** Levels of serum lactate collected from treatment-naïve OAC patients (*n* = 33) were correlated with the levels of circulating cytokines determined by multiplex ELISA assays. Significant correlations indicated with a star, and the size of the circle reflects the magnitude of the correlation value R. The R values are depicted in the lower portion of the corrograms for significant correlations only. Correlation matrixes depicted in (**D**) and (**E**) show the correlations between the expression of TNF-α in CD4^+^ and CD8^+^ cells with IC expression on CD4^+^ and CD8^+^ cells, with the significant correlations summarised in (**F**) and (**G**), respectively. Blue depicts positive correlations, and red depicts negative correlations. Spearman correlation analysis was used

The effect of acidosis on anti-tumour and pro-tumour cytokine profiles of OAC-derived T cells was investigated to acquire a deeper understanding of the potential immunosuppressive effects of acidosis in the context of OAC (Fig. 5).

Acidosis did not significantly alter the frequency of IFN-γ-producing T cells, IL-4-producing T cells or IL-10-producing T cells ex vivo (Fig. S5). Acidosis also did not significantly alter the cytotoxic potential of CD8^+^ T cells demonstrated by no significant alteration in CD107a expression (Fig. S5D). However, the frequency of TNF-α-producing CD4^+^ T cells was significantly decreased in pH 5.5 cRPMI compared with cells cultured in pH 6.6 and pH 7.4 cRPMI (Fig. 5A, B). Likewise, the frequency of TNF-α-producing CD4^+^ T cells was significantly decreased in pH 6.6 cRPMI compared with cells cultured in pH 7.4 cRPMI (Fig. 5A, B). Similarly, the frequency of TNF-α-producing CD8^+^ T cells was significantly decreased in pH 5.5 cRPMI compared with cells cultured in pH 6.6 and pH 7.4 cRPMI (Fig. 5A, B). The frequency of TNF-α-producing CD8^+^ T cells was significantly decreased in pH 6.6 cRPMI compared with cells cultured in pH 7.4 cRPMI (Fig. 5A, B). Furthermore, levels of serum lactate from treatment-naïve OAC patients negatively correlated with circulating levels of pro-inflammatory cytokine IL-6 (Fig. 5C). The expression of TNF-α by CD4^+^ cells positively correlated with TIGIT and CD160 expression on the surface of CD4^+^ cells (Fig. 5D, F). Within the CD8^+^ T cell compartment, the expression of TNF-α by CD8^+^ cells positively correlated with CD160 and KLRG-1 expression and negatively correlated with LAG-3 and CTLA-4 expression on the surface of CD8^+^ cells (Fig. 5E, G).

**Fig. 6:**
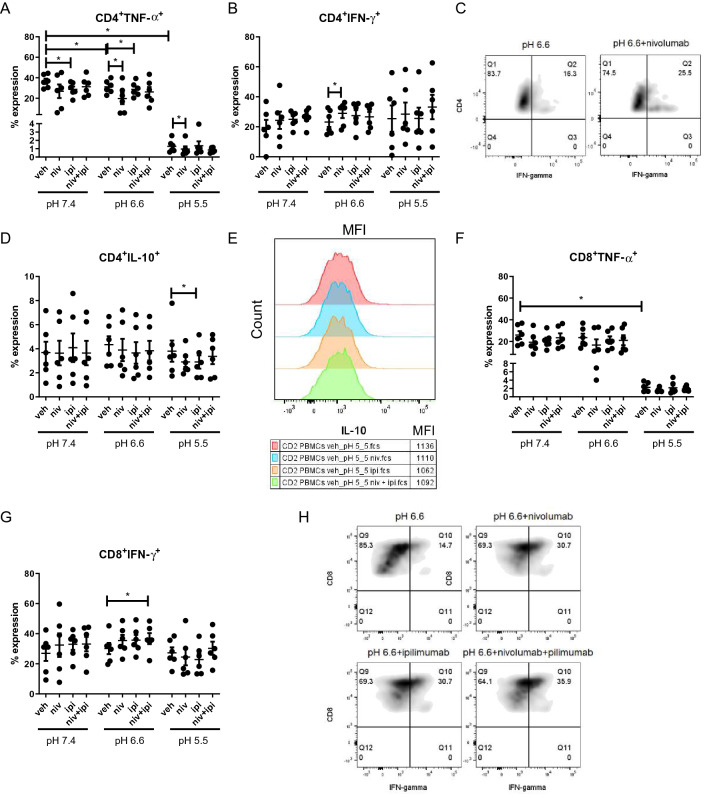
ICB increases IFN-γ production under moderately acidic conditions only and decreases IL-10 production by T cells under acidic conditions. PBMCs were isolated from peripheral blood of treatment-naïve OAC patients (*n* = 6) and expanded ex vivo for 7 days in the presence of plate bound anti-CD3/anti-CD28 and recombinant human IL-2. Following a 7-day expansion, PBMCs were cultured for 48 h in media with increasing levels of acidity (pH 7.4, pH 6.6 and pH 5.5) in the absence or presence of ICB. ICB included nivolumab (niv), ipilimumab (ipi) or dual nivolumab-ipilimumab (niv + ipi). Intracellular staining was conducted to assess CD3^+^CD4^+^ and CD3^+^CD8^+^ cell production of TNF-α, (**A** and **F**), IFN-γ (B-C and G-H) and IL-10 (**D**–**E**) cytokines by flow cytometry. Non-significant changes are outlined in Fig. S5. Paired, non-parametric t test, *p < 0.05. Expression presented as percentages ± SEM

Use of ICB to enhance anti-tumour cytokine profiles is an attractive therapeutic approach to boost response rates for OAC patients; however, the ability of ICB to promote anti-tumour cytokine profiles under acidic conditions is of utmost importance. Therefore, the effect of ICB on anti-tumour and pro-tumour cytokine profiles under acidic conditions reflective of the acidic tumour microenvironment was assessed (Fig. 6). Single-agent nivolumab significantly decreased the production of TNF-α in CD4^+^ T cells compared with untreated cells in pH 6.6 cRPMI and pH 5.5 cRPMI (Fig. 6A). Similarly, single-agent ipilimumab significantly decreased the production of TNF-α in CD4^+^ T cells compared with untreated cells in pH 6.6 cRPMI only but not in pH 5.5 cRPMI or pH 7.4 cRPMI (Fig. 6A). Interestingly, single-agent nivolumab significantly increased the production of IFN-γ in CD4^+^ T cells compared with untreated cells in pH 6.6 cRPMI culture conditions only (Fig. 6B, C). Furthermore, single-agent ipilimumab significantly decreased the production of IL-10 in CD4^+^ T cells compared with untreated cells in pH 5.5 cRPMI only but not in pH 7.4 or pH 6.6 cRPMI (Fig. 6D, E). ICB did not significantly alter the frequency of IL-4-producing T cells compared with untreated cells in pH 7.4, pH 6.6 or pH 5.5 cRPMI (Fig. S5).

Single-agent nivolumab or ipilimumab treatment did not significantly enhance IFN-γ expression by CD8^+^ T cells; however, dual nivolumab and ipilimumab significantly increased the production of IFN-γ in CD8^+^ T cells compared with untreated cells in pH 6.6 cRPMI only (*p* = 0.03) but not in pH 7.4 cRPMI or pH 5.5 cRPMI (Fig. 6G, H). Single agent or dual ICB did not significantly alter the production of TNF-α, IL-4 or IL-10 in CD8^+^ T cells ex vivo under pH 7.4, 6.6 or 5.5 cRPMI (Fig. S5). However, single-agent ipilimumab treatment significantly decreased the degranulation of CD8^+^ T cells compared with untreated cells in pH 5.5 cRPMI (*p* = 0.03) but had no effect in pH 6.6 cRPMI or pH 7.4 cRPMI (Fig. S5).

Overall, acidosis significantly decreased the percentage of TNF-α-producing T cells ex vivo*.* ICB increased T cell production of IFN-γ under moderately acidic conditions (pH 6.6) but not severe acidic conditions (pH 5.5) and decreased IL-10 production by T cells under severe acidic conditions only.

### Serum lactate levels positively correlate with circulating levels of soluble LAG-3 and soluble Tie-2 in OAC patients

**Fig. 7:**
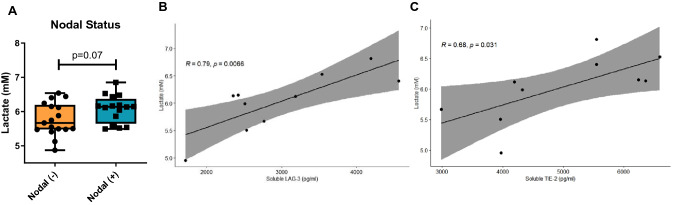
Levels of circulating lactate positively correlate with soluble levels of LAG-3 and Tie-2 in the serum of OAC patients. Serum lactate levels were assessed in OAC patients post-treatment, pre-operatively at time of surgical resection of tumour using a lactate assay in duplicate technical replicates (*n* = 33). Serum lactate levels displayed based on negative (*n* = 17) versus positive nodal status (*n* = 16) (**A**) and based on the presence of adverse features (nodal metastasis, lymphovascular invasion, perineural invasion and serosal invasion) (**B**). Correlation plots demonstrating the significant positive correlations between serum lactate levels and circulating soluble LAG-3 levels (**C**) and circulating soluble Tie-2 levels (**D**) (*n* = 10). Mann–Whitney test to compare between 2 groups and Pearson correlation tests were used, *p < 0.05

Studies have shown that serum lactate levels and tumoural expression of lactate dehydrogenase A and B have prognostic value in several cancer types [6]. Therefore, this study assessed the levels of circulating lactate levels in a cohort of 33 OAC patients and interrogated the prognostic value of serum lactate levels in oesophageal cancer. The levels of circulating lactate in the serum of OAC patients ranged from 4.87 to 7.28 mM with a mean value of 5.97 ± 0.08 mM in a cohort of 33 OAC patients post-treatment (Fig. 7). Serum lactate levels were correlated with tumour stage and clinical outcomes in a cohort of 33 OAC patients post-treatment. However, serum lactate levels did not significantly correlate with tumour stage or clinical outcomes (data not shown). Similarly, analysis of a cohort of 80 oesophageal cancer patients available from the TCGA showed that levels of lactate dehydrogenase A and lactate dehydrogenase B were not associated with overall survival or tumour progression-free survival (data not shown). A recent publication from our group identified nodal metastasis as a superior prognostic indicator and predictive factor for patient survival compared with pathologic treatment response. Therefore, we assessed if patients who had the presence of nodal metastasis had higher levels of circulating serum lactate. Interestingly, patients with nodal metastasis had higher levels of serum lactate compared with patients whose tumours did not metastasise to lymph nodes (*p* = 0.07) (Fig. 7A). Circulating lactate levels in OAC patients did not significantly correlate with the frequency of circulating T cells expressing ICs, T cell activation markers and specific T cell differentiation states for a cohort of 10 OAC patients (Fig. S6A–C).

Given the important pleiotropic role of lactate in suppressing T cells, enhancing DC maturation, promoting angiogenesis and tumour progression, serum lactate levels from a subcohort of consecutive OAC patients undergoing oesophagectomy in St. James’s Hospital were correlated with circulating levels of a panel of 54 pro-inflammatory, pro-angiogenic, immunostimulatory and immunosuppressive molecules in a cohort of 10 OAC patients [17, 18]. Interestingly, serum levels of lactate in OAC patients positively correlated with circulating levels of soluble LAG-3 (*r* = 0.79 and *p* = 0.006, Fig. 7B) and the pro-angiogenic factor Tie-2 (*r* = 0.68 and *p* = 0.03, Fig. 7C).

## Discussion

A large body of evidence in the literature indicates that protons present in the tumour microenvironment as a result of tumour acidification are recognised as a damage-associated molecular pattern by DCs and subsequently enhance DC maturation and function [1]. However, the opposite has been proven for the effect of acidosis on lymphocyte function, which has been shown to suppress IFN-γ production by Th1 cells and CD8^+^ T cell cytolytic ability [12], inducing an anergic state [13]. Our results support the hypothesis that acidity suppresses anti-tumour T lymphocyte function demonstrated by a significant upregulation of inhibitory immune checkpoints TIM-3, LAG-3, and CTLA-4 on the surface of OAC-derived T cells when cultured under low pH conditions ex vivo. These findings offer a fundamental biological connection between acidosis-like conditions and IC biology of T cells. Expression of CTLA-4 [19], TIM-3 [20] and LAG-3 [21] ICs is typically upregulated on dysfunctional and/or exhausted T cells. This further supports the hypothesis that acidic conditions suppress T cell function perhaps through immune checkpoint-intrinsic signalling and their blockade may enhance T cell function under acidic conditions. The upregulation of this particular set of ICs also provides an exploitable therapeutic target that could be harnessed via co-blockade of all three ICs. Clinical trials will be required to assess the potential dose-limiting side effects of this trio combination.

It is important to note that the efficacy of CTLA-4 blockade is limited to a subpopulation of patients, and development of acquired resistance is common [22]. This has prompted the investigation into additional immune checkpoints such as TIM-3 and LAG-3 [23] which are highly expressed on Tregs at sites of tissue inflammation and were significantly upregulated on the surface of OAC-derived T cells cultured under acidic conditions in this study [24]. Findings by Deng et al. highlighted the importance of LAG-3 in suppressing anti-tumour immune responses, whereby LAG-3 blockade reduced tumour growth, potentiated anti-tumour responses of CD8^+^ T cells and decreased the population of immunosuppressive cells in a murine model of head and neck squamous cell carcinoma [21]. Similarly, extensive data in preclinical cancer models [25–27] and in vitro cultures of patient samples [28] demonstrate that TIM-3 blockade enhances anti-tumour T cell responses, particularly in conjunction with PD-1 blockade.

CTLA-4 ICB in preclinical murine models of colon adenocarcinoma significantly decreased tumour growth, increased the frequency of tumour-infiltrating effector T cells and selectively depleted intra-tumoural regulatory T cells [29]. Although CTLA-4 ICB-mediated depletion of tumour-infiltrating regulatory T cells has not been observed in human cancers such as melanoma, prostate and bladder cancers, an increase in tumour-infiltrating CD4^+^ and CD8^+^ cells has been reported [30]. Complementary findings from our study demonstrated that ipilimumab treatment significantly decreased production of IL-10 by CD4^+^ T cells under severe acidic conditions ex vivo. Moreover, dual nivolumab and ipilimumab treatment significantly enhanced the production of IFN-γ by CD8^+^ T cells under moderately acidic conditions (pH 6.6), but this effect was lost under severe acidic conditions (pH 5.5). Suggesting that dual nivolumab and ipilimumab treatment might be a promising intervention to limit the immunosuppressive effects of the acidic tumour microenvironment. Of note, dual nivolumab and ipilimumab treatment failed to enhance the production of IFN-γ by CD8^+^ T cells under severe acidic conditions (pH 5.5) highlighting that extratumoural acidity might represent a mechanism of resistance to ICB. Addition of ICBs to target ICs that were upregulated under severe acidity may be necessary to overcome treatment resistance to CTLA-4 and PD-1 ICB. TIM-3 and LAG-3 were also upregulated under severe acidity and may represent mechanisms of acquired resistance to PD-1 and CTLA-4 inhibitors and co-blockade with additional ICBs such as TIM-3 or LAG-3 may be necessary. Clinical trials will be useful in this regard to identify synergistic ICB combinations. Complementary studies in the literature further support the immunosuppressive effects of tumour acidity on the efficacy of ICB. Interestingly, combining oral bicarbonate therapy to neutralize tumour acidity in combination with anti-CTLA-4 or anti-PD1 therapy improved antitumor responses in multiple models, including complete remissions in some subjects [14]. Taken together with the findings from this study, a rationale is highlighted for raising intratumoural pH through oral buffer therapy to improve responses to ICB, with the potential for immediate clinical translation for treating OAC patients.

Moreover, single-agent nivolumab and ipilimumab significantly decreased the production of tumour-promoting cytokine TNF-α under neutral pH conditions and acidic conditions. TNF-α possesses pleiotropic effects in promoting angiogenesis, tumour-promoting inflammation and resistance to tumour cell apoptosis [31]. In Lewis Lung murine models, knock down of the TNF-α receptor (TNF-R2) in cancer cells promotes robust anti-tumour effects upon administration of low dose murine TNF-alpha, whereas in wild-type mice, it enhanced tumour growth [32]. However, under severe acidic conditions only (pH 5.5) single-agent ipilimumab significantly decreased the cytotoxic potential of CD8^+^ T cells; however, this effect was not observed under neutral pH conditions or moderately acidic conditions (pH 7.4 and pH 6.6, respectively). Under severe acidic conditions, single-agent ipilimumab also decreased the frequency of terminally differentiated effector memory T cells and promoted their differentiation into central memory T cells which may reflect the observed decrease in cytotoxic potential of CD8^+^ T cells as terminally differentiated effector memory T cells are highly cytotoxic and rapidly release their effector molecules, whereas central memory T cells are not as specialised in cytolytic function and acquire effector functions less rapidly [33–36].

We also found that systemic lactate levels positively correlated with circulating levels of soluble LAG-3 receptor. Although expression of LAG-3 on T cells promotes T cell dysfunction, the soluble version of LAG-3 was a good prognostic marker in gastric cancer and positively correlated with CD8^+^ T cell frequency and secretion of IL-12 and IFN-γ in peripheral blood [37]. Moreover, a study by Fougeray et al*.* [38] identified soluble LAG-3 protein as an immunopotentiator for therapeutic vaccines. Soluble LAG-3 binds to MHC class II inducing maturation of monocyte-derived DCs in vitro and is used as a vaccine adjuvant to induce CD4 Th1 and CD8 T cell responses in vivo [38]. This raises an important question about whether lactate might be regulating the cleavage of membrane-bound LAG-3 on the surface of immune cells to enhance DC maturation. Given the large body of evidence in the literature indicating that acidic conditions promote DC maturation via binding of protons to ASIC receptors on DCs [11], overall acting as a damage-associated molecular pattern, lactate-mediated generation of soluble LAG-3 may represent a novel mechanism to synergistically promote DC maturation. Further research will be required to test this hypothesis.

Our study reaffirms the already established link between lactate and angiogenesis in which we found that systemic lactate levels correlated with an important pro-angiogenic mediator Tie-2. Lactate has been found to bind to Tie2 when it is expressed as a cell surface receptor on endothelial cells activating the PI3K/Akt pathway [39]. The function of soluble Tie-2 in the cancer setting remains unknown; however, soluble levels of Tie-2 were upregulated in patients with Crohn’s disease and ulcerative colitis compared with healthy controls and correlated with more advanced stage disease [40]. Similarly, soluble levels of Tie-2 were elevated only in periphery artery disease patients that were the most angiogenically compromised (critical limb ischaemia) [41]. Angiogenesis plays an important role in mediating metastatic dissemination, and interestingly, our study identified that patients with nodal metastasis had higher levels of circulating lactate compared with patients who did not have nodal metastasis, establishing lactate as a potential adverse prognostic indicator in OAC patients. Several studies have identified that circulating lactate levels were indicative of a poor prognosis in other cancer types, but this is the first study to demonstrate the prognostic utility of serum lactate [6]. Collectively, these findings establish lactate as not only a promoter of immune evasion in OAC but also of other key hallmarks of cancer including promotion of pro-angiogenic processes and metastasis.

Taken together, our results provide preclinical evidence for the immunosuppressive effects of extracellular pH on T lymphocyte function in the context of OAC and offer a rationale supporting the use of ICB in particular for ipilimumab to boost anti-tumour T cell immunity to help limit the immunosuppressive effects of tumoural acidity in OAC. In addition, novel ICs TIM-3 and LAG-3 are also highlighted as potential therapeutic targets to combine with PD-1 or CTLA-4 ICBs under acidic conditions to boost anti-tumour immunity.

## Supplementary Information

Below is the link to the electronic supplementary material.

Supplementary file1 (DOCX 3933 kb)
