# Supplementary material for: Acidosis significantly alters immune checkpoint expression profiles of T cells from oesophageal adenocarcinoma patients
Source: Cancer Immunol Immunother. 2022 Jun 16;72(1):55–71. doi: 10.1007/s00262-022-03228-y (PMC9813044; doi:10.1007/s00262-022-03228-y)
Supplement: Supplementary file 1 — Supplementary file1 (DOCX 3933 kb) [file 262_2022_3228_MOESM1_ESM.docx]

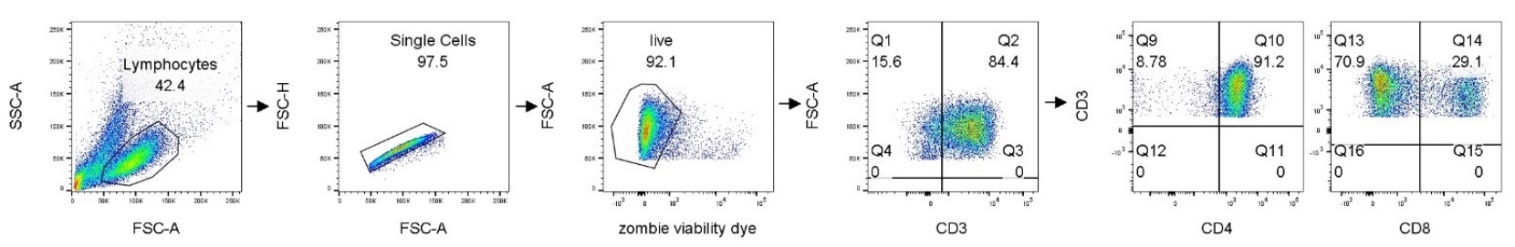
**
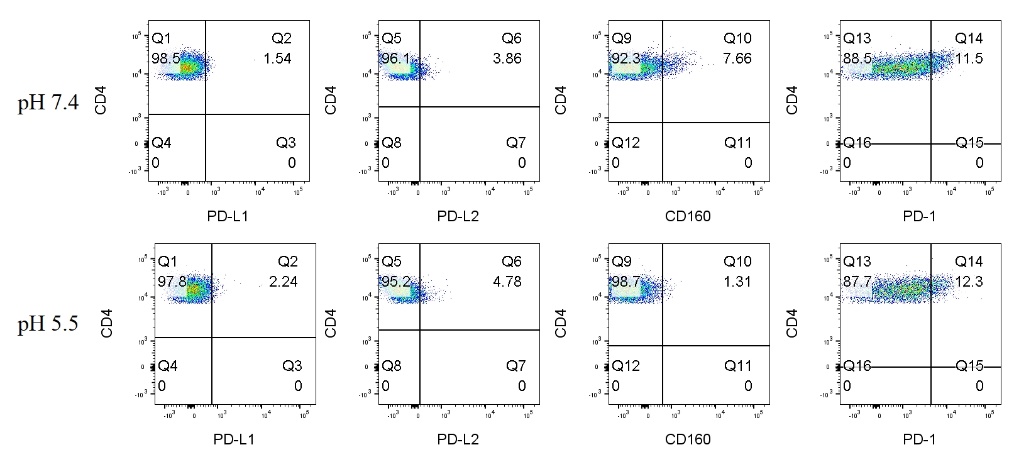

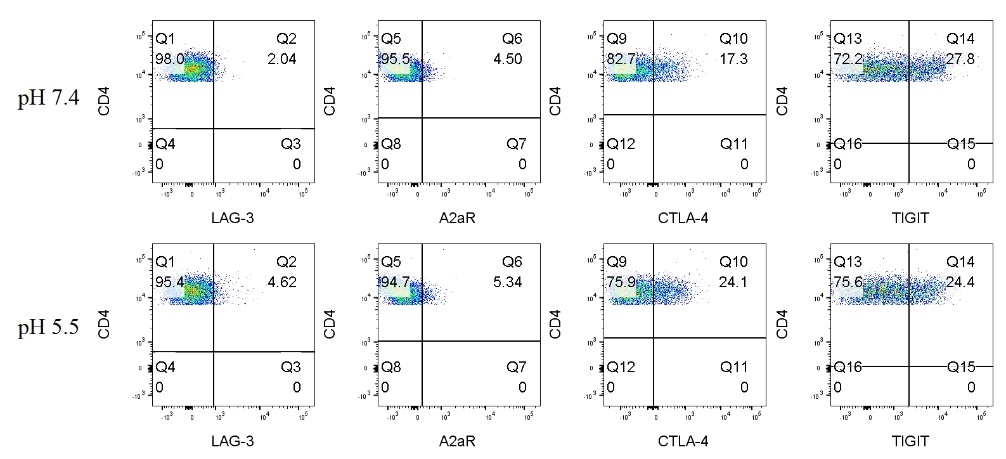

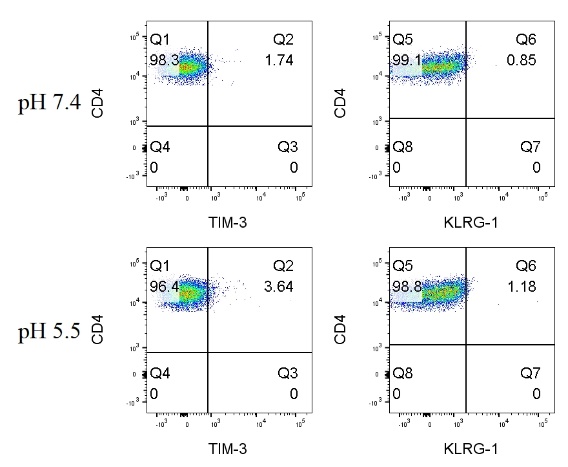
**Supplemental

**Figure S1:** **Gating strategy for assessing expression of ICs on T cells surfaces by flow cytometry.** Gate 1 included all cells in the FSC versus SSCA plot, doublet cells were then excluded using FSC-H versus FSC-A plot, dead cells were excluded using zombie viability dye. The surface expression of PD-L1, PD-L2, CD160, PD-1, TIM-3, KLRG-1, LAG-3, A2aR, CTLA-4 and TIGIT was assessed on CD3^+^CD4^+^ cells and CD3^+^CD8^+^ cells. Representative dot plots are shown for each gated on CD3^+^CD4^+^ cells following 48h culture of PBMCs in pH 7.4 cRPMI and pH 5.5 cRPMI.

**
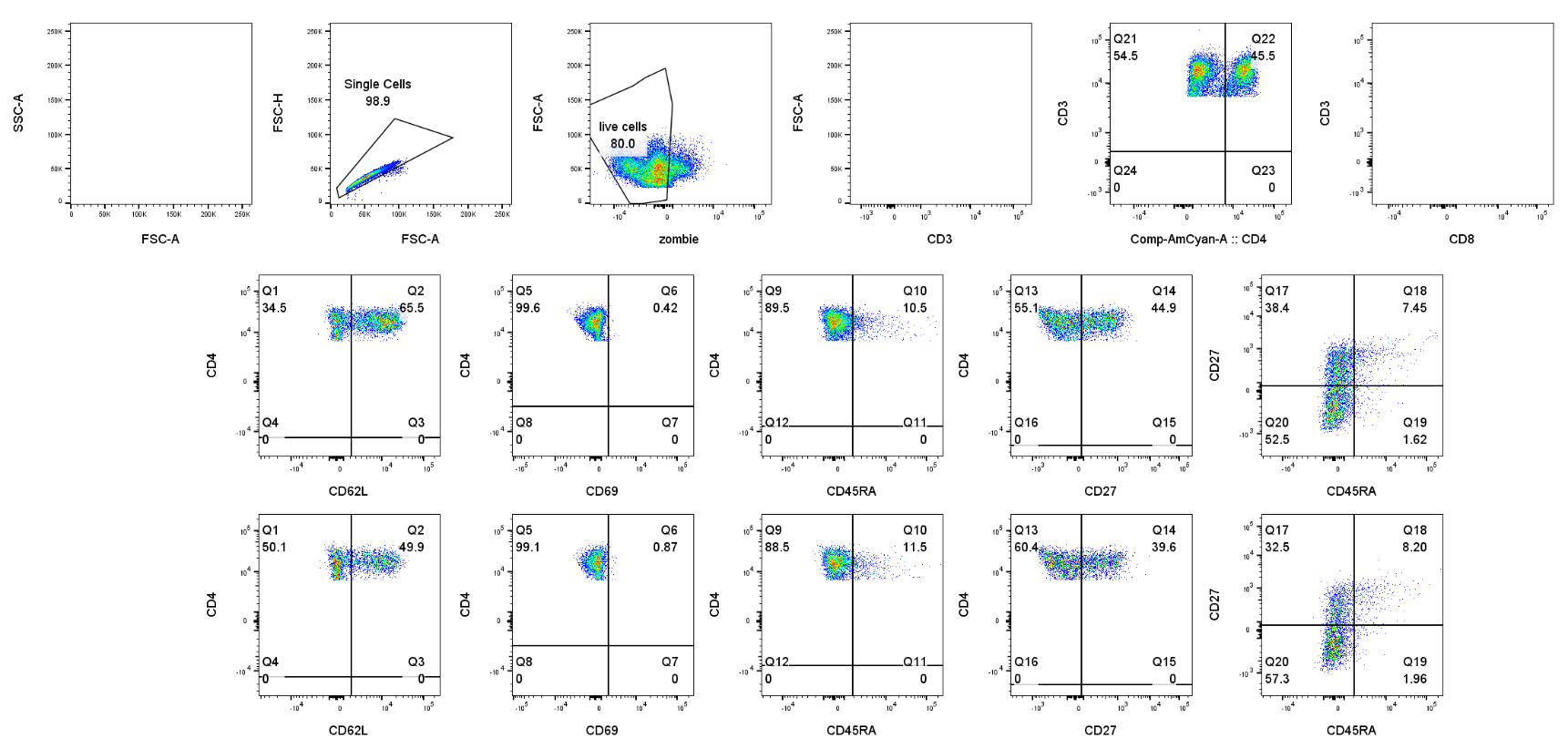

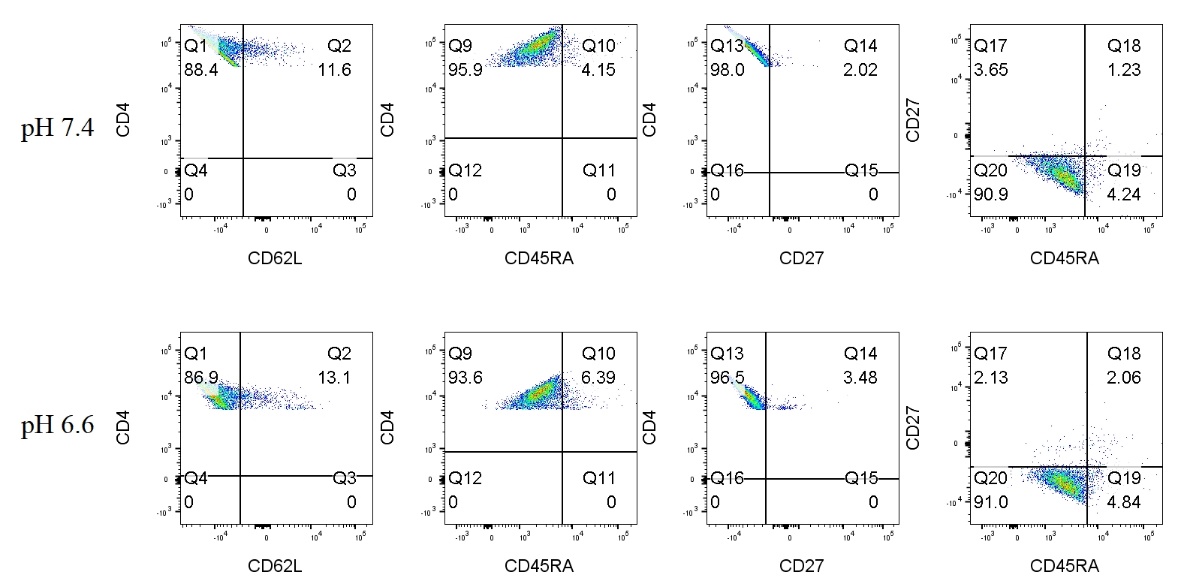

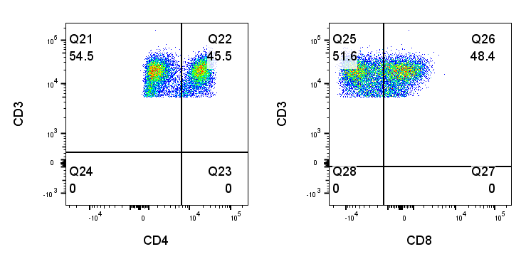
**
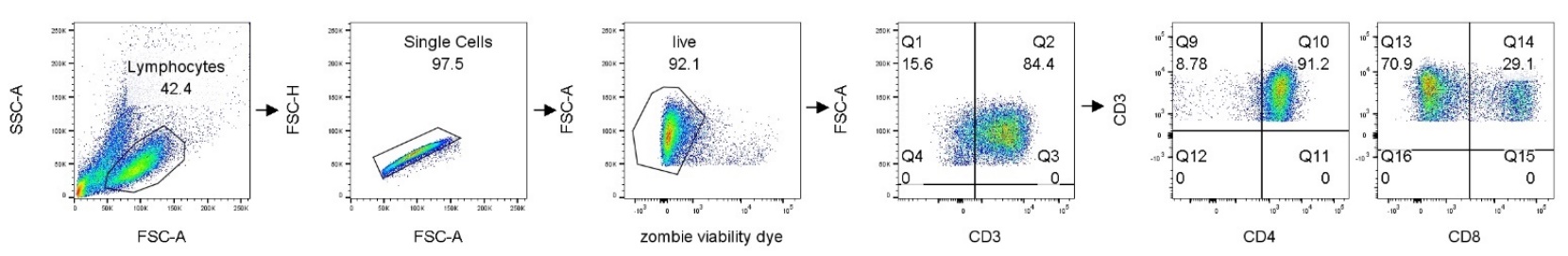


**Figure S2: Gating strategy for assessing expression of T cell activation markers and T cell differentiation states by flow cytometry.** Gate 1 included all cells in the FSC versus SSCA plot, doublet cells were then excluded using FSC-H versus FSC-A plot, dead cells were excluded using zombie viability dye. The surface expression of CD62L, CD69, CD45RA and CD27 as well as T cell differentiation states was assessed on CD3^+^CD4^+^ cells and CD3^+^CD8^+^ cells. Representative dot plots are shown for each marker gated on CD3^+^CD4^+^ cells following 48h culture of PBMCs in pH 7.4 cRPMI and pH 5.5 cRPMI. Representative dot plots also shown depicting viable naïve (CD27^+^CD45RA^+^), central memory (CD27^+^CD45RA^-^), effector memory (CD27^-^CD45RA^-^) and terminally differentiated effector memory (CD27^-^CD45RA^+^) CD3^+^CD4^+^ cells.

**Figure S3: Effect of acidic conditions on the co-expression of ICs on the surface of OAC patient-derived T cells.** PBMCs were isolated from peripheral blood of treatment-naïve OAC patients (n=6) and expanded *ex vivo* for 7 days in the presence of plate bound anti-CD3/anti-CD28 and recombinant human IL-2. Following a 7-day expansion, PBMCs were cultured for 48h in media with increasing levels of acidity (pH 7.4, pH 6.6 and pH 5.5). CD3^+^CD4^+^ and CD3^+^CD8^+^ cells were stained with zombie viability dye and antibodies specific for IC ligands and receptors and co-expression of multiple ICs was assessed by flow cytometry. Non-significant changes are shown in A-E. Significant changes are outlined in **Figure 2**. Expression presented as percentages ± SEM. Paired, non-parametric t test for PBMCs (n=6) and paired parametric t-test for OE33 cells (n=3).

**Figure S4: Effect of acidosis on activation marker expression in the absence and presence of ICB.** PBMCs were isolated from peripheral blood of treatment-naïve OAC patients (n=6) and expanded *ex vivo* for 7 days in the presence of plate bound anti-CD3/anti-CD28 and recombinant human IL-2. Following a 7-day expansion, PBMCs were cultured for 48h in media with increasing levels of acidity (pH 7.4, pH 6.6 and pH 5.5) in the absence or presence of ICB. ICB included nivolumab (niv), ipilimumab (ipi) or dual nivolumab-ipilimumab (niv + ipi). Expression of markers reflective of T cell activation status was assessed on viable CD3^+^CD4^+^ and CD3^+^CD8^+^ cells by flow cytometry (n=6). Markers assessed included: CD62L, CD69, CD27 and CD45RA. The percentage of viable naïve (CD45RA^+^CD27^+^), central memory (CD45RA^-^CD27^+^), effector memory (CD45RA^-^CD27^-^) and terminally differentiated effector memory (CD45RA^+^CD27^-^) CD3^+^CD4^+^and CD3^+^CD8^+^cells was also determined by flow cytometry. Only non-significant changes are shown in this figure and the significant changes are outlined in **Figure 3** and **Figure 4**. Dead cells were excluded using a zombie viability dye. Paired, non-parametric t test, *p<0.05. Expression presented as percentages ± SEM.

**Figure S5: Effect of acidity on T cell production of cytokines in the absence and presence of ICB.** PBMCs were isolated from peripheral blood of treatment-naïve OAC patients (n=6) and expanded *ex vivo* for 7 days in the presence of plate bound anti-CD3/anti-CD28 and recombinant human IL-2. Following a 7-day expansion, PBMCs were cultured for 48h in media with increasing levels of acidity (pH 7.4, pH 6.6 and pH 5.5). Intracellular staining was conducted to assess CD3^+^, CD3^+^CD4^+^ and CD3^+^CD8^+^ cell production of IFN-γ (**A**), IL-10 (**B**) and IL-4 (**C**) cytokines by flow cytometry. The cytotoxic potential of CD3^+^CD8^+^ T cells was also assessed using a CD107a degranulation assay by flow cytometry (**D**). To assess effect of acidity on the ability of ICB to alter cytokine profiles – following a 7-day T cell activation protocol PBMCs were cultured for 48h in media with increasing levels of acidity (pH 7.4, pH 6.6 and pH 5.5) in the absence or presence of ICB. ICB included nivolumab (niv), ipilimumab (ipi) or dual nivolumab-ipilimumab (niv+ipi). Intracellular staining was conducted to assess CD3^+^CD4^+^ cell production of IL-4 (E) and CD3^+^CD8^+^ cell production of IL-10 (F) and IL-4 (G) cytokines by flow cytometry. CD3^+^CD8^+^ cell cytotoxicity was also assessed in the absence and presence of ICB (H). Only non-significant changes are shown in the figure and significant changes are outlined in **Figure 5** and **Figure 6**. Paired, non-parametric t test, *p<0.05. Expression presented as percentages ± SEM.

**Figure S6: Corograms illustrating correlation values between serum lactate levels in OAC patients and the frequency of circulating T cells expressing ICs, T cell activation markers and specific T cell differentiation states.** Significant correlations indicated with a star, the size of the circle reflects the magnitude of the correlation value R. The R values are depicted in the lower portion of the corograms for significant correlations only. Blue depicts positive correlations and red depicts negative correlations. Spearman correlation analysis used.

**Supplemental Table 1: Flow cytometry panels.**

| **Immune checkpoint panel 1** | **Immune checkpoint panel 1** | **Immune checkpoint panel 1** | **Activation marker panel 1** | **Cytokine panel 1** | **Cytokine panel 2** |
| --- | --- | --- | --- | --- | --- |
| LAG-3-FITC | PD-L1-FITC | TIM-3-ViobrightFITC | CD62L-FITC |  |  |
| A2aR-PE | PD-L2-PE |  | CD69-PE | IL-10-PE | CD107a-PE |
| CTAL-4-PE/Cy5 | CD160-PerCPCy5.5 | CD3-PerCPCy5.5 | CD8-PerCP | CD3-PerCPCy5.5 | CD3-PerCPCy5.5 |
| TIGIT-PE/Cy7 | PD-1-PE/Cy7 |  | CD45RA-PE/cy7 | IL-4-PE/Cy7 |  |
| CD8-BV421 | CD8-BV421 | CD8-BV421 | Zombie Violet | CD8-BV421 | CD8-BV421 |
| CD4-BV510 | CD4-BV510 | CD4-BV510 | CD4-BV510 | CD4-BV510 | IFNγ-BV510 |
| CD3-APC | CD3-APC | KLRG-1-APC | CD3-APC | TNF-α-APC | CD4-APC |
| Zombie NIR | Zombie NIR | Zombie NIR | CD27-APC/Cy7 | Zombie NIR | Zombie NIR |
